# Understanding expertise and non-analytic cognition in fingerprint discriminations made by humans

**DOI:** 10.3389/fpsyg.2014.00737

**Published:** 2014-07-16

**Authors:** Matthew B. Thompson, Jason M. Tangen, Rachel A. Searston

**Affiliations:** School of Psychology, The University of QueenslandSt Lucia, QLD, Australia

**Keywords:** expertise, fingerprints, decision making, forensics, non-analytical reasoning, testimony, instance-based learning

As a novice in a particular domain, the cognitive feats that experts are capable of performing seem impressive, even extraordinary. According to the well-established exemplar theory of categorization (e.g., Brooks, 1987; Medin and Ross, 1989), a new category member in everyday classification (e.g., a bird, a table, or a car) or expert classification (e.g., an abnormal chest x-ray, a patient with myocardial ischaemia, or a poor chess move) is categorized on the basis of its similarity to individual prior cases. Often this sensitivity develops effortlessly and without any intention to learn similarities or differences among the exemplars.

Experts can do a lot with a little. Across various domains of expertise, it seems that experts can perform quickly and accurately when given only a small amount of information, as in chess (Gobet and Charness, 2006); fireground command (Klein, 1998); radiology (Myles-Worsley et al., 1988; Evans et al., 2013), and dermatology (Norman et al., 1989). The experiential knowledge based on the hundreds of thousands of prior instances serves as a rich source of analogies to permit efficient problem solving.

A fruitful approach to understanding these cognitive feats has been to understand where expertise lies in various domains. Expertise in ball sports, for example, seems to lie in anticipating where the ball will be (Abernethy, 1991); expertise in wine seems to lie in applying verbal labels (Hughson and Boakes, 2001); expertise in radiology seems to lie in rapid discrimination of normal and abnormal radiographs (Evans et al., 2013); and expertise in chess seems lie in rapid retrieval of board configurations from memory (Chase and Simon, 1973).

Over the last several years, we have been working with a fascinating group of experts who spend several hours a day examining a highly structured set of impressions. When a fingerprint is found at a crime scene it is a human examiner, not a machine, who is faced with the task of identifying the person who left it. Professional fingerprint examiners are usually sworn police officers who use image enhancement tools, such as Photoshop or a physical magnifying glass, and database tools to provide a list of possible matching candidates. They place a crime scene print and a suspect print side-by-side—physically or on a computer screen—and visually compare the prints to judge whether the prints came from the same person or two different people.

These fingerprint examiners have testified in court for over one hundred years, but there have been few experiments directly investigating the extent to which experts can correctly match fingerprints to one another, how competent and proficient fingerprint experts are, how examiners make their decisions, or the factors that affect performance (Loftus and Cole, 2004; Saks and Koehler, 2005; Vokey et al., 2009; Spinney, 2010b; Thompson et al., 2013a). Indeed, many examiners have even claimed that fingerprint identification is infallible (Federal Bureau of Investigation, 1984). Academics, judges, scientists, and US Senators have reported on the absence of solid scientific practices in the forensic sciences. They highlight the absence of experiments on human expertise in forensic pattern matching, suggesting that faulty analyses may be contributing to wrongful convictions of innocent people (Edwards, 2009; National Research Council, 2009; Campbell, 2011; Carle, 2011; Expert Working Group on Human Factors in Latent Print Analysis, 2012; Maxmen, 2012), and they lament the lack of a research culture in the forensic sciences (Mnookin et al., 2011). The field of forensics is, however, beginning to acknowledge the central role that fallible humans play in the identification process (Tangen, 2013).

Our first point of inquiry was to see whether qualified, court practicing fingerprint examiners are any more accurate than the person on the street, and to get a feel for the kinds of errors examiners make. In our first experiment (Tangen et al., 2011), we tested the matching accuracy of fingerprint examiners from Australian state and federal law enforcement agencies. In a signal detection paradigm, we created ground-truth matching prints for use as targets, and highly-similar, nonmatching prints from a national database search for use as distractors. We found that qualified, court-practicing fingerprint experts were exceedingly accurate compared with novices. Experts tended to err on the side of caution by making more errors of the sort that could allow a guilty person to escape detection than errors of the sort that could falsely incriminate an innocent person. A similar experiment, with participants from the US Federal Bureau of Investigation, produced similar results (Ulery et al., 2011), and a follow-up experiment found variability in the consistency within and between examiners' decisions (Ulery et al., 2012). An examiner's expertise seems to lie, not in matching prints *per se*, but in discriminating highly similar but nonmatching prints (Thompson et al., 2013a).

In a follow-up experiment (Thompson et al., 2013b), we replicated (Tangen et al., 2011) but with genuine crime scene matching prints from casework (where the ground truth is uncertain), and with the addition of two trainee participant groups. Intermediate trainees—despite their lack of qualification and average 3.5 years' experience—performed about as accurately as qualified experts who had an average 17.5 year's experience. It appears that people can learn to distinguish matching from similar nonmatching prints to roughly the same level of accuracy as experts after a few years of experience and training. New trainees—despite their 5-week, full-time training course or their 6 months experience—were not any better than novices at discriminating matching and similar nonmatching prints, they were just more conservative. It appears that early training and/or experience may not necessarily result in more accurate judgments, but may simply result in a more conservative response bias. Again we concluded that the superior performance of experts was a result of their ability to identify the highly similar, but nonmatching fingerprints as such.

What the findings mean for reasoning about expert performance in the wild is an open question (e.g., Koehler, 2008, 2012; Mnookin, 2008; Thompson et al., 2013a), but these findings do contradict the notion that fingerprint identification is infallible, and that the fingerprint identification “methodology” can be disembodied from a judgment about whether two fingerprints match or not.

Our second point of inquiry was to understand the nature of expertise in fingerprint matching—to understand where a fingerprint examiner's expertise lies. Through experience and feedback, an expert can rapidly retrieve, from memory, previous instances and decisions relevant to the current situation, whereas novices rely more on formal rules and procedures (Brooks, 2005; Norman et al., 2007). Fingerprint examiners claim that careful, deliberate analysis is the basis of the work that they do (Busey and Parada, 2010), but a hallmark of genuine expertise is the ability to accurately perform a domain relevant task quickly (Kahneman and Klein, 2009). Do fingerprint examiners rely on the same non-analytic cognitive processes as experts in other domains of expertise?

Busey et al. (2011) found that experts move their eyes differently from novices, and Busey and Vanderkolk (2005) found that experts performed better than novices at identifying the matching fragments of fingerprints in noise after a short delay. They also found that inverted fingerprints produced a delayed N170 event-related potential response in experts but not in novices, suggesting that experts process upright fingerprints configurally (Busey and Parada, 2010).

In a series of experiments, we further examined the nature of fingerprint expertise (Thompson, 2014). We added artificial noise to all the print pairs, and inverted half and kept the other half upright, and found that experts could discriminate prints even when the prints were highly noisy. Unexpectedly, fingerprint experts did not show the classic inversion effect seen in face recognition. We tested the short term memory of experts and novices by separating fingerprint pairs in time by a few seconds, and found that experts were better than novices at discriminating print, and that experts were far better at discriminating similar, nonmatching prints. We tested the long term memory of experts and novices by asking them to learn a set of fingerprints to be recognized a few minutes later, but found no difference in long term memory accuracy between experts and novices, and both groups performed around the level of chance. We tested the ability of experts and novices to discriminate prints by presenting them briefly on screen, and found that experts could accurately discriminate prints when presented for just 2000 ms, and the largest difference between experts and novices was on similar, nonmatching prints. We then further reduced the stimuli presentation time, and found that experts were more accurate than novices overall, and that experts had a much better idea about whether a pair of prints match or not in a rapid period of time. With such short presentation times (i.e., from 250 to 2000 ms), there is little time to engage in careful, deliberate analysis of the minutiae in a fingerprint image in order to make accurate decisions.

These findings suggest that fingerprint experts are capable of making accurate decisions when the amount of visual information in the prints is dramatically decreased. It is clear that, through experience, experts can learn the regularities of matching and nonmatching prints, and rapidly compare new prints to memory in order to make accurate judgments. The findings above are in stark contrast to the common and consistent claims in formal training, textbooks, and courtroom testimony: that fingerprint identification is a “scientific process” that requires careful, thorough analysis in order for judgments to be accurate.

Fingerprint expertise is particularly interesting because of the sheer amount of experience that examiners have with the stimuli. Their full-time job, often in departments that run 24 hours a day, is to visually compare crime scene prints to suspect and database candidates. It's difficult to imagine any other domain in which there is so much attention and exposure to a highly constrained stimulus, where the task is to definitively report that two images come from the same, single source or not. And the stakes are high: innocent people could be wrongly convicted, and guilty people could be wrongly acquitted.

This vast experience allows experts to resolve information in a print: to correctly regard ambiguous information that is more consistent with within-source variability as a “match,” and correctly regard ambiguous information that is more consistent with between-source variability as a “non-match.” An ambiguous mark on a fingerprint, for example, can be regarded as signal (i.e., as evidence of a “match”), or it can be disregarded as noise (i.e., as evidence of a “non-match”). This kind of process is undoubtedly operating in novices too, but the ambiguity cannot be sufficiently resolved unless the examiner has accumulated enough matching and nonmatching exemplars in memory to point to one direction or the other. One clear result of this vast experience is the experts' capacity to disregard, to “see through” the ambiguity and surface structure of similar prints and discriminate them accurately. We think that further study of the nature of fingerprint expertise will inform general theories for the development of expertise, while also providing an empirical basis for claims made by expert witnesses in the courtroom.

## Conflict of interest statement

The authors declare that the research was conducted in the absence of any commercial or financial relationships that could be construed as a potential conflict of interest.
